# Genome-wide study on the polysomic genetic factors conferring plasticity of flower sexuality in hexaploid persimmon

**DOI:** 10.1093/dnares/dsaa012

**Published:** 2020-06-17

**Authors:** Kanae Masuda, Eiji Yamamoto, Kenta Shirasawa, Noriyuki Onoue, Atsushi Kono, Koichiro Ushijima, Yasutaka Kubo, Ryutaro Tao, Isabelle M Henry, Takashi Akagi

**Affiliations:** 1 Graduate School of Environmental and Life Science, Okayama University, Okayama 700-8530, Japan; 2 Kazusa DNA Research Institute, Kisarazu, Chiba 292-0818, Japan; 3 Institute of Fruit Tree and Tea Science, NARO, Hiroshima 739-2494, Japan; 4 Graduate School of Agriculture, Kyoto University, Kyoto 606-8502, Japan; 5 Department of Plant Biology and Genome Center, University of California, Davis, CA 95616, USA

**Keywords:** flexible sexuality, monoecy, polyploid, GWAS

## Abstract

Sexuality is one of the fundamental mechanisms that work towards maintaining genetic diversity within a species. In diploid persimmons (*Diospyros* spp.), separated sexuality, the presence of separate male and female individuals (dioecy), is controlled by the Y chromosome-encoded small-RNA gene, *OGI*. On the other hand, sexuality in hexaploid Oriental persimmon (*Diospyros kaki*) is more plastic, with *OGI*-bearing genetically male individuals, able to produce both male and female flowers (monoecy). This is thought to be linked to the partial inactivation of *OGI* by a retrotransposon insertion, resulting in DNA methylation of the *OGI* promoter region. To identify the genetic factors regulating branch sexual conversion, genome-wide correlation/association analyses were conducted using ddRAD-Seq data from an F_1_ segregating population, and using both quantitative and diploidized genotypes, respectively. We found that allelic ratio at the Y-chromosomal region, including *OGI*, was correlated with male conversion based on quantitative genotypes, suggesting that *OGI* can be activated in *cis* in a dosage-dependent manner. Genome-wide association analysis based on diploidized genotypes, normalized for the effect of *OGI* allele dosage, detected three fundamental loci associated with male conversion. These loci underlie candidate genes, which could potentially act epigenetically for the activation of *OGI* expression.

## 1. Introduction

Sexuality is a fundamental mechanism that acts for the maintenance of genetic diversity within a species. In contrast to animals, hermaphroditism is thought to be ancestral and most common in flowering plants. A minority of species have subsequently, and independently, evolved separated sexuality, with separate male and female individuals (dioecy).^1^^,^^2^ These species represent up to 5% of angiosperm species.^3^^,^^4^ Plant biologists have thrived to understand the evolutionary steps associated with transitions into or out of dioecy since the first findings of genetic sex determination in flowering plants.^5^ Typical transitions out of dioecy have been associated with domestication events in some crops. For example, in papaya^6^ or grape,^7^ the loss of function of the Y chromosome-encoded female suppressor genes, resulting in hermaphroditism, was artificially selected, presumably for stable cultivation. On the other hand, polyploidization is also thought to be a main key player in the evolution of sexuality, often causing a transition out of dioecy.^8^^,^^9^

**Figure 1:**
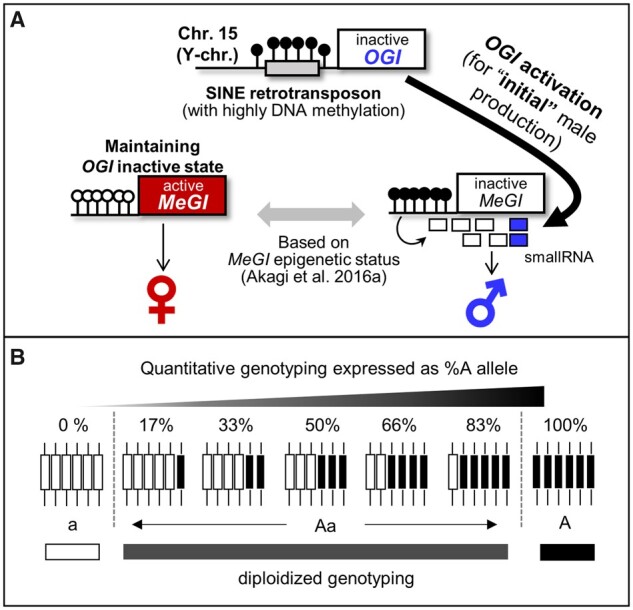
Approaches used to identify the genetic factors regulating *OGI* activity. (A) Model of the male flower formation in monoecious hexaploidy *D. kaki* individuals. *OGI* is inactivated by the presence of a highly methylated SINE-like retrotransposon in the promoter region.^11^*OGI* is occasionally expressed and triggers small-RNA production by *MeGI*. The resulting DNA methylation in the promoter region represses *MeGI* expression to switch to the production of male flowers. After production of the initial male flowers, the fate of flower sexuality depends on maintenance or release of DNA methylation on *MeGI*.^11^ (B) Two genotyping approaches are available for polyploid populations, that tailor to two different models for the effect of genetic inheritance: (i) in the additive model, heterozygous alleles are further characterized based on their copy number, (ii) in the diploidized additive model, only diploidized genotypes are considered (homozygous or heterozygous), irrespective of the ratio of alleles in the heterozygous states.

In dioecious diploid persimmon species (*Diospyros spp.*), the Y chromosome-encoded small-RNA gene *OGI* is responsible for expression of maleness, through the repression of its autosomal counterpart, a gene called *MeGI*.^10^ Importantly, hexaploid cultivated Oriental persimmon (*Diospyros kaki*) has evolved a more plastic sex determination system from the dioecious system, possibly through epigenetic regulation of *OGI* and *MeGI*.^11^^,^^12^ In hexaploid Oriental persimmon, genetically male individuals, carrying at least one copy of Y-chromosome (including *OGI*), exhibit monoecy, where both male and female flowers are produced by the same tree, although they often produce only female flowers.^13^ This monoecious system is based on the semi-inactivation of *OGI* by the presence of a highly methylated retrotransposon insertion, named *Kali*, within the *OGI* promoter region, and on the variable DNA methylation of the *MeGI* sequence (Fig. 1A).^11^ With the exception of a few genetically female cultivars, which do not carry the *OGI* sequence but occasionally bear male flowers,^13^^,^^14^ this male production is consistent with the change of epigenetic regulation on *MeGI.*^15^ Therefore, the *OGI/MeGI* system is thought to be conserved as the regulator of sex determination in hexaploid persimmon as in diploid persimmon, despite the fact that expression of *OGI* in hexaploid persimmon has never been formally observed, possibly because it is extremely restricted both temporally and spatially.

In hexaploidy persimmon trees, the morphological difference between male and female developing buds is first visible in June. Typically, all floral buds produced on a branch develop similarly, either into male or female flowers. The first flowering year, all branches bear female flowers. In subsequent years, female branches tend to produce female branches but there is a low frequency of transition from female to male. Once a branch is male, it typically produces male branches in subsequent years, but there is a low frequency of reversal to femaleness as well. These observations are consistent with the hypothesis that occasional activation of *OGI* expression results in the accumulation of small-RNA and -DNA methylation on *MeGI*, leading to the production of male flowers. Subsequently, maintenance of maleness is dependent on the maintenance of the DNA methylation status of *MeGI*.^11^ According to this hypothesis, the frequency of *OGI* activation should be directly associated with male conversion. Here, we used segregating sibling plants that carry the *OGI* gene to investigate the genetic factors that regulate male conversion in hexaploid Oriental persimmon.

Investigating the genetic architecture of traits with multiple alleles and mixed inheritance patterns in polyploid species is challenging.^16^ Genotyping in polyploids is complicated by the possibility of more than two alleles at each locus, and the existence of different heterozygous states. For instance, in the case of autohexaploid persimmon with hexasomic inheritance,^17^ we can define five heterozygous states; AAAAAa (5:1), AAAAaa (4:2), and AAAaaa (3:3) AAaaaa (2:4) to Aaaaaa (1:5). Quantitative genotyping of such loci using qPCR is a possible solution,^17^^,^^18^ but it is time-consuming. Recent advances in sequencing and genotyping technologies now allow calling high-density single nucleotide polymorphism (SNP), and accurate determination of allele ratio and allele dosage for polyploid genomes. Genome-wide association analysis, using quantitative genotypes, coupled with realistic genetic models^19^^,^^20^ could shed light on the genetic basis of complex traits.

In this study, to examine the genetic factors underlying female-to-male conversion in hexaploid Oriental persimmon, we collected high-density genotype information in a segregating population exhibiting biases in male flower ratio. Considering the nature of polysomic inheritance, we developed genome-wide correlation/association analyses for polyploid persimmon using two different models (Fig. 1B): (i) an additive model using quantitative genotypes in the form of allelic ratio, and (ii) a diploidized additive model using diploidized genotypes.^21^ The results led to the identification of genetic regions and candidate genes potentially involved in the regulation of female-to-male conversion, providing novel insights into the genetic basis of flexible sexuality after adaptation to polyploidization or domestication.

## 2. Materials and methods

### 2.1. Plant materials and selection of individuals carrying *OGI* alleles

**Figure 2:**
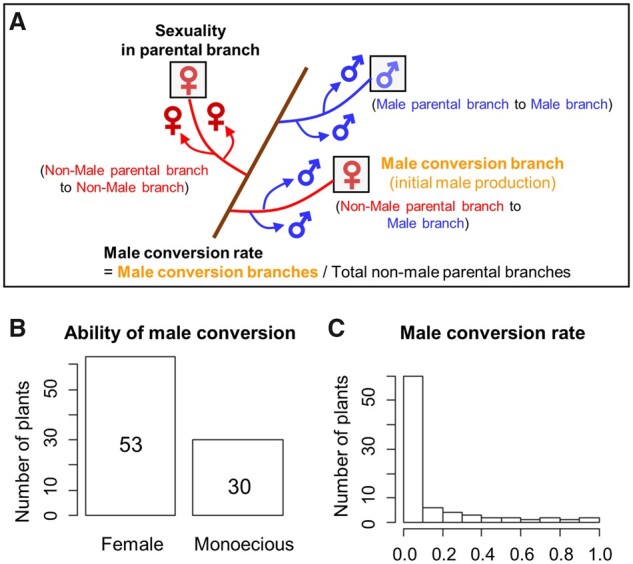
Phenotypic diversity in the YTF_1_ population. (A), Schematic representation of flowering branches in hexaploidy persimmon. Male conversion rate was calculated as the numbers non-male parental branches (female or previously non-flowering) producing male flowers, divided by the total number of non-male parental branches. A male conversion branch was defined as a non-male parental branch producing male flowers on at least one branch. Distribution of phenotypic values in the 83 individuals that carried at least one copy of the *OGI* locus: cumulative ability of male conversion (over 5 years) (B) and male conversion rate (C).

The segregating F_1_ population produced from a cross between ‘Yamatogosho’ (6 A + XXXXXX) × ‘Taishu’ (6 A + XXXXYY), named the YTF_1_ population, was developed in 2009, and exhibited variation in the frequency of male production since the first flowering in 2013–14. Flower sexuality from a total of 5,016 non-male (female or no flower) parental branches was assessed for 5 years, from 2014 to 2018. A male conversion branch was defined as a non-male parental branch producing male flowers on at least one branch (Fig. 2A). Two phenotypes were recorded each year. ‘Ability of male conversion’, reflects the presence or absence of male flowers on any given branch. In other words, if any branch in a tree had switched to male that year, the tree was scored as 1 for this phenotype. If none of the branches had converted, it was scored as 0. The second phenotype, ‘Male conversion rate’, was calculated by dividing the number of branches that converted to male in a specific year, by the total number of non-male parental branches. Both phenotypes were scored each year. To identify the genetic factors regulating male conversion, a total 83 individuals, each carrying at least one *OGI* allele were selected. To identify individuals carrying the *OGI* genes, genomic DNA was extracted from leaves of each individual using the CTAB method,^13^ and *OGI* was amplified by PCR using two primer pairs (Supplementary Table S1), with a programme of 95°C for 3 min followed by 32 cycles of 95°C for 20 s, 56°C for 15 s, and 72°C for 1 min.

### 2.2. Genome-wide genotyping

ddRAD-sequencing libraries were prepared according to previous reports^22^ using 107 individuals of the YTF_1_ population and their two parents ‘Yamatogosho’ and ‘Taishu’. The libraries were sequenced on an Illumina HiSeq4000 platform, and generating PE100 reads at the Vincent J. Coates Genomics Sequencing Laboratory, University of California Berkeley. The reads were aligned to the reference genome of *Diospyros lotus*, a wild relative close to Oriental persimmon (http://persimmon.kazusa.or.jp/index.html)^23^ using the Burrows–Wheeler Aligner with default parameters [version 0.7.12 (https://github.com/lh3/bwa)^24^; Supplementary Table S2]. Based on these parameters, it is possible that some allelic and potential paralogous polymorphisms were occasionally derived from non-specific mapping. The resulting sam files were converted to bam files and subsequently vcf format using bcf/vcftools^25^ and Varscan.^26^ For genome-wide association/correlation analyses, only the 83 individuals carrying the *OGI* allele were used (Supplementary Table S2). Individual genotypes were only considered if coverage > 20. A total of 95,639 heterozygous markers were selected by using bcftools with the following options: minor allele frequency within the population > 0.05 and data were available for at least 50% of the individuals. For detection of transmission ratio distortion, only the 91 individuals of the YTF_1_ population with total coverage > 200 Mb were used, to decrease the possibility of detecting the false transmission distortion when using low coverage data (Supplementary Table S2). A total of 39,344 loci were selected, with average coverage between 60 and 200 for each of the 91 individuals, minimum coverage in the parents > 60, and by using bcftools with the following options: minor allele frequency > 0.01 and max-missing = 1. The coverage threshold was used to filter uniquely mapped sequences. Transmission distortion ratios of alternative alleles were calculated as the log value of [allele ratio in the YTF_1_/allele ratio in the parents]. Here, for the ‘allele ratio in the parents’ values, we calculated the alternative allelic read coverages in the two parents independently, and averaged them. Significant transmission distortion was detected by standardizing the transmission distortion ratios using z-transformation with the threshold of *P*-value < 1E-10.

### 2.3. Heritability estimation

Direct calculation of broad-sense heritability was difficult because our data include many missing values. Alternatively, we used estimated narrow-sense heritability whose genetic effect was approximated by an additive genetic relationship matrix calculated from genome-wide diploidized genotype. To estimate narrow-sense heritability (${\hat{h}}^{2}$), we estimated the genetic and error variance components (${\hat{\sigma }}_{g}^{2}$ and ${\hat{\sigma }}_{e}^{2}$) with a restricted maximum likelihood (REML) approach,^27^ because the replication of phenotypic observations was insufficient to calculate these components using the standard ANOVA method. For the REML approach, the phenotypic variance V was defined by the following equation:

(1) $$V=A{\hat{\sigma }}_{g}^{2}+I{\hat{\sigma }}_{e}^{2},$$

where A is a genetic relationship matrix between individuals and I is an *n *×* n* identity matrix. In the genetic relationship matrix, the element $A_{jk}$ was defined as:

(2) $$A_{jk}=\;\sum_{i=1}^{m}\left(x_{ij}-{2p}_{i})(x_{ik}-{2p}_{i}\right)/{2p}_{i}(1-p_{i}),$$

where $x_{ij}$ (coded as 0, 1, 2) is the number of copies of the reference allele for the *i*th SNP of the *j*th individual, $p_{i}$ is the minor allele frequency for the *i*th SNP, and m is the total number of markers. The REML solution of Equation (1) was obtained by using the function ‘mixed.solve’ in the R package *rrBLUP* version 4.4.^28^ The estimated variance components were used to calculate heritability with the following equation:

(3) $${\hat{h}}^{2}\;=\;{\hat{\sigma }}_{g}^{2}/({\hat{\sigma }}_{g}^{2}\;+\;{\hat{\sigma }}_{e}^{2}).$$

### 2.4. Genome-wide correlation/association analyses using additive and diploidized additive models

Allele composition (or quantitative genotype) at each SNP locus was estimated from the frequency of alternative alleles in mapped reads. Genome-wide correlation analysis was conducted using a total 40,111 of the 95,639 SNPs, in which duplex–pentaplex genotypes were predicted in at least 23/83 YTF_1_ individuals (<28% individuals with at least one recessive allele). To test if a locus affected male conversion in a dosage-dependent manner, Pearson product moment correlation analysis was conducted between the ratio of alternative alleles at each locus and the phenotype values of each individual. The coefficient values were standardized using z-transformation, to estimate *P*-values. Significant association loci were detected with the threshold of −log(*P*-value) > 5.60, representing a Bonferroni-corrected significance threshold of 0.1.

Diploidized genotype at each locus was defined with the threshold of 5% (and 95%) of alternative alleles in the mapped reads to call heterozygosity. Genome-wide association analysis was conducted using the R package rrBLUP, with a linear mixed model (LMM).^28^ Significant association loci were detected with the threshold of −log(*P-*value) > 5.98, representing a Bonferroni-corrected significant threshold of 0.1. Linkage disequilibrium (LD) surrounding the loci highly associated with the sex expression (*P* < 10E^−6^) was evaluated using squared Pearson’s correlation coefficient (*r*^2^) with the −*r*^2^ command in the software PLINK version 1.07.

### 2.5. Genome-wide analyses compensated with the effect of *OGI* allele dosage

Allele dosage of *OGI* was estimated with quantitative PCR using LightCycler 480 (Roche Diagnostics, Mannheim, Germany), according to a previous report^13^ (Supplementary Table S1 for primers). Briefly, using primer sets for *OGI* and the control locus, *L5R*, quantitative PCR analyses were conducted using THUNDERBIRD SYBR qPCR Mix (TOYOBO, Osaka, Japan). For six individuals of the YTF_1_ population, due to the lack of DNA, allele dosage on Chr. 15, the peak of 17,052,751-bp on the sex-chromosome was used as a proxy for *OGI* allele dosage (around 18.12-MB on Chr. 15).^23^

To assess the effect of loci on the male conversion traits independently of the effect of *OGI* allele dosage, which substantially affect male production ability, ‘partial correlation’ analysis was conducted, based on quantitative genotypes. The partial correlation, considering the effect of *OGI* allele dosage, assumes the following equation:

(4) $$y=\frac{\left(r_{by}-r_{ay}\times r_{ab}\right)}{\sqrt{1-{r_{ay}}^{2}}\times \sqrt{1-{r_{ab}}^{2}}}$$

where *r_by_* is the correlation coefficient between the quantitative genotype at the locus and the phenotype; *r_ay_* is the correlation coefficient between *OGI* allele dosage and the phenotype; and *r_ab_* is the coefficient between *OGI* allele dosage and the quantitative genotype at the locus. The partial correlation coefficient values were standardized with z-transformation to estimate *P*-values.

For the diploidized additive model with diploidized genotypes, we assumed the following LMM:

(5) $$y=\beta_{o}O+Zg+S\tau +\varepsilon$$

where *y* is a vector of the phenotype; *β_o_* is a fixed effect for *OGI* allele dosage (*O*) and *Z* is an incident matrix relating *y* to *g*. The variable *g* models the genetic background of each line as a random effect with Var[*g*] = *Kσ*^2^_g_, where *σ*^2^_g_ is the genetic variance. *K* is an additive kinship matrix calculated from the genotype data. *S* is a vector of genotype of each polymorphic site. The variable *τ* models the additive SNP effect. *ε* is a matrix of the residual effects with Var[*ε*] = *Iσ*^2^_e_, where *I* is an identity matrix and *σ*^2^_e_ is the residual variance. To solve the mixed model, we used functions in the R package rrBLUP.^28^ The additive kinship matrix *K* was calculated using the ‘A.mat’ function. The variance components *σ*^2^_g_ and *σ*^2^_e_ were estimated using the ‘mixed.solve’ function. *P*-values for the SNP markers were calculated using the ‘GWAS’ function, which we slightly modified to use quantitative values of *OGI* allele dosage as a fixed effect.

## 3. Results and discussion

### 3.1. Characterization of flower sex expression in the YTF_1_ population

Within the YTF_1_ population, 53 of 83 individuals only produced female flowers despite carrying at least one copy of the *OGI* gene (Fig. 2B and Supplementary Table S3). The monoecious individuals (*N* = 30) showed a wide range of male conversion rate, i.e. conversion from non-male (female and no flowers) parental branches producing male flowers within the 5 years assessment (Fig. 2A and C). As suggested in previous reports,^13^^,^^29^ the frequency of male flowers substantially increased as years went by, during the assessment (average male conversion rate are 0.07, 0.13, and 0.15 during the first 3 years; Supplementary Fig. S1 and Supplementary Table S3). Most of the male parental branches also produced male flowers in subsequent years (Supplementary Table S4), which was hypothetically due to maintenance of epigenetic marks (or DNA methylation) on *MeGI* in the parental branches.^11^ Consistent with these results, the estimated narrow-sense heritability for male conversion rate, across 5 years, was 0.28. Therefore, we assessed the cumulative ability to produce male branches from female branches throughout the 5 years, to compensate for the limited number of replicates, and reflect the effect of environmental inputs.

### 3.2. No large-scale transmission distortion in the YTF_1_ population

**Figure 3:**
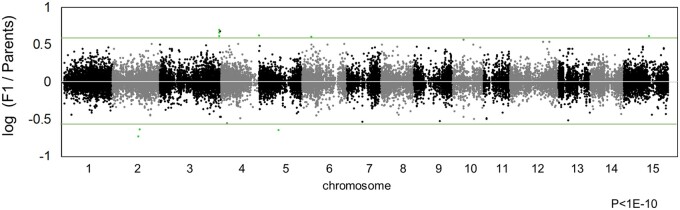
Genome-wide transmission distortion of allelic ratios compared with the parental alleles. Dynamic (or fragmental) transmission ratio distortions were not detected. A value of ‘0’ for the log [F_1_/parents] (white line) is expected from complete lack of transmission distortion. Green lines indicated the threshold (*P* = 1E-10, see Section 2).

Plants, which have recently polyploidized, often show unbalanced chromosome/genome compositions, such as frequent aneuploidy or unstable inheritance patterns,^18^^,^^30^^,^^31^ as also observed in hexaploid Oriental persimmon.^17^^,^^32^ The distribution of the alternative allele ratios per individual of the YTF_1_ population delineated seven peaks, putatively corresponding to nulliplex to hexaplex genotypes in each individual (Supplementary Fig. S2A). The distribution of mean alternative allele frequency in the YTF_1_ population as a whole and at each SNP loci, exhibited 13 potential peaks, putatively corresponding to 13 allelic combinations in an hexaploidy F_1_ cross (Supplementary Fig. S2B). We documented transmission ratio distortion throughout the genome, in the YTF_1_ population, but did not detect any large-scale distortion in allele ratio from the expected values based on the observed parental genotypes (Fig. 3). For example, our observed inheritance of *OGI* was consistent with mendelian heredity pattern of *OGI* in the progeny of a cross between a nulliplex and a duplex individual (χ^2^ test *P* = 0.62). Although some SNP loci exhibited significant transmission distortion (*P *<* *1E-11), other loci within the same haploblocks did not exhibit this distortion (Supplementary Table S5). We assumed that these loci were incorrectly genotyped due to ambiguity in parental genotyping with no replicates.

### 3.3 The Y chromosome contributes to male conversion in a dosage-dependent manner

**Figure 4:**
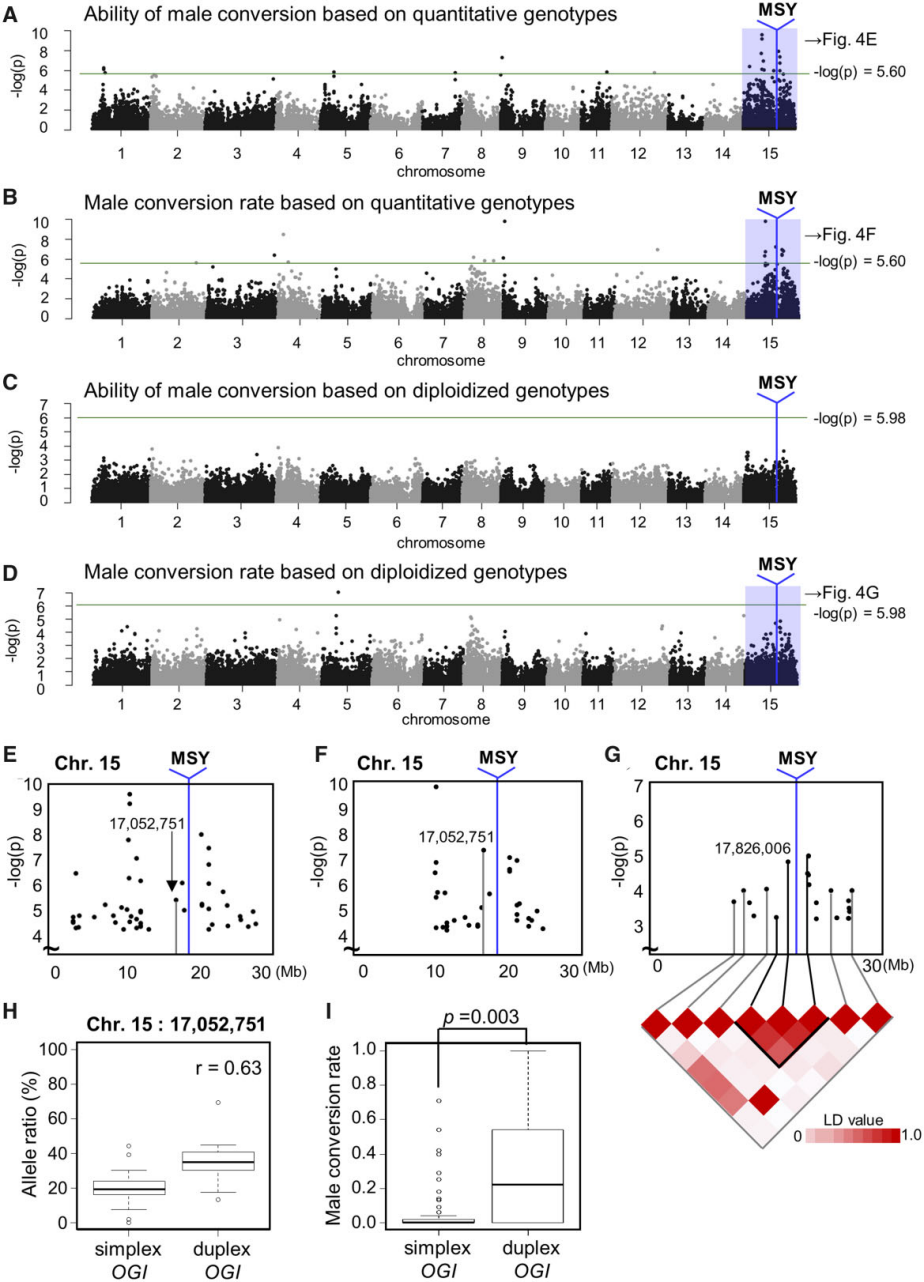
Genome-wide correlation/association analysis for male productivity. Genome-wide correlation analysis using quantitative genotypes for the ability of male conversion (A) and male conversion rate (B) traits. Genome-wide association analysis using diploidized genotypes, for the ability of male conversion (C) and male conversion rate (D) traits. The sex-chromosome (Chr. 15) is highly association with both ability of male conversion and male conversion rates. The blue vertical lines correspond to the MSY, including the *OGI* locus, which is not included in the reference genome of the wild diploid species (*D. lotus*).^23^ The green lines indicate −log(*P*-value) > 5.60 for the additive model, and > 5.98 for the diploidized additive model, respectively, representing the Bonferroni-corrected threshold of 0.1. (E-G) Close-up of the association loci on the sex chromosome, including the MSY region, for ability of male conversion (E) and male conversion rate (F) based on quantitative genotypes, and for male conversion rate based on diploidized genotypes (G). For (G), the MSY is contained within the peak locus. (H) Correlation between allele ratio at the peak (17,052,751-bp) on the sex chromosome (Chr. 15, see E and F), and *OGI* allele dosage, as detected by qPCR. (I) Association between male conversion rate and the *OGI* allele dosage. The individuals carrying duplex *OGI* alleles exhibited significantly higher male conversion rates (*t*-test *P*-value shown).

Genome-wide correlation analyses based on quantitative genotypes using an additive model resulted in several peaks, the strongest of which was located on the sex-chromosome (Chr. 15).^23^ This was true both for the ability of male conversion and the male conversion rate traits (Fig. 4A and B). Although the male-specific region of the Y-chromosome (MSY) is not included in the reference pseudomolecule (DLO_r1.0.pseudomolecule), it has been genetically anchored within Chr. 15 (around 18.12 Mb).^23^ Two highly associated peaks flanked the MSY that includes *OGI* (Fig. 4E and FSupplementary Table S6A and B). Some loci within the sex chromosome commonly showed significant correlation with the ability of male conversion and the male conversion rate (Fig. 4E and F), suggesting that these two traits were regulated by the same allele combinations. When using the diploidized additive model based on diploidized genotypes, the region surrounding the MSY showed a weak association to male conversion rate (Fig. 4C and D and Supplementary Table S6C). The location of the haploblock including MSY, based on diploidized genotypes (Fig. 4G), was consistent with the peaks based on quantitative genotypes (Fig. 4E and F).

*OGI* allele numbers, as determined by quantitative PCR, were highly correlated with the numbers of alleles in the nearest peak produced using the additive model (*r* = 0.63, 78.2% of individuals exhibited identical quantitative genotypes, Fig. 4H and Supplementary Table S7), and with male conversion abilities (Fig. 4I;*P* = 0.003 for male conversion rate with Student *t*-test, Supplementary Table S8; *P* = 0.004 for ability of male conversion). Multiple regression tests with the loci quantitatively associated with male conversion abilities indicated that, amongst these loci, *OGI* exhibited the strongest effect (*P* < 0.0002, Supplementary Table S9). These results supported the idea that the *OGI* locus significantly contributes to male conversion ability in a dosage-dependent manner, and suggested that *cis*-elements within or close to *OGI* can play important roles for the activation of *OGI*. This concept is similar to the dosage sex-chromosome systems in *Rumex* and *Humulus*,^33^ where dioecious sex determination is dependent on X/autosome balance. In these systems, individuals with intermediate sexuality, bearing hermaphrodite flowers, also appear in association with irregular X/autosome ratios between 0.5 and 1.0.^33^

### 3.4. Identification of candidate loci activating *OGI*

**Figure 5:**
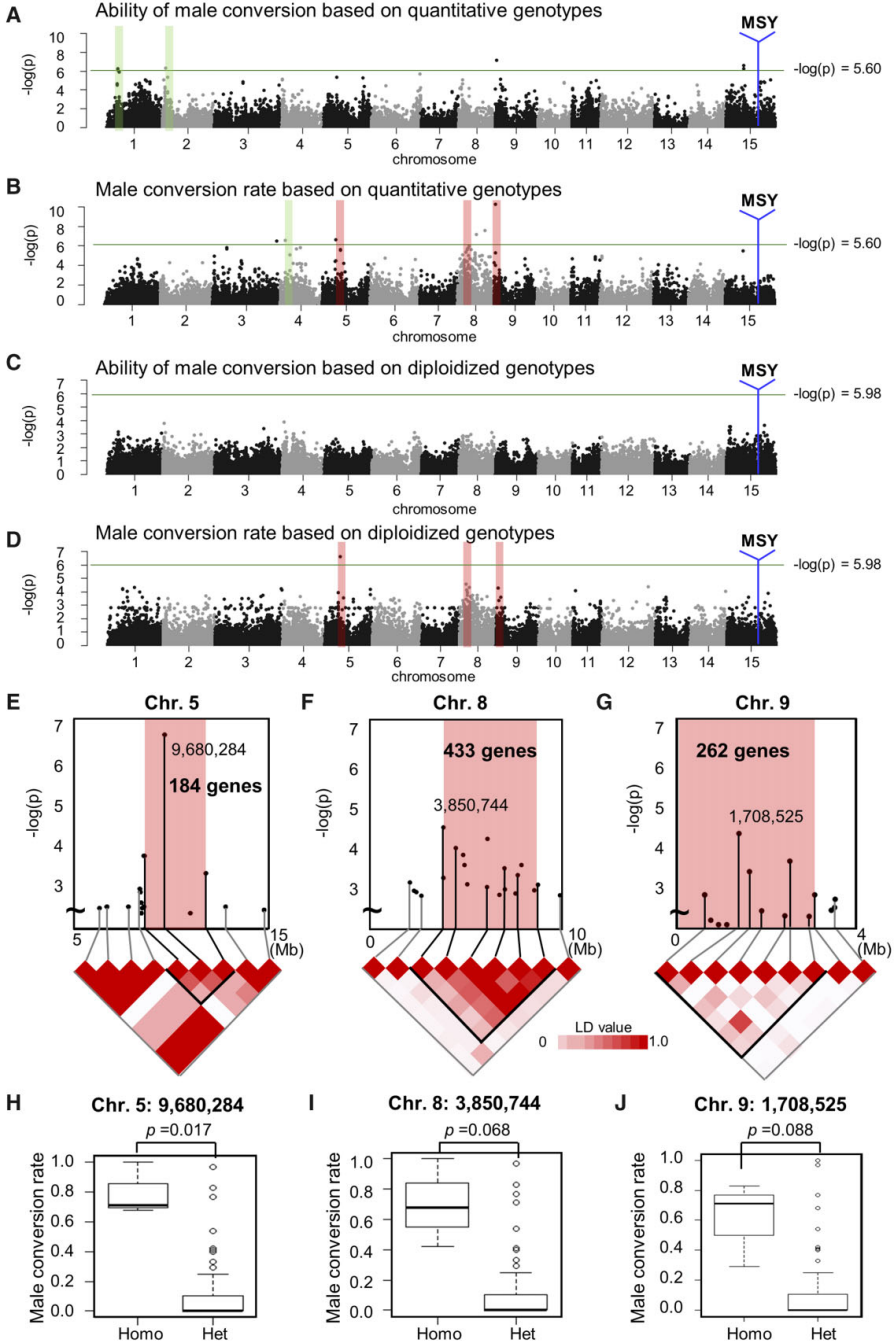
Genome-wide correlation/association analysis after normalization for the effect of *OGI* allele dosage. Genome-wide partial correlation analysis of the ability of male conversion (A) and male conversion rate (B), using quantitative genotypes in the additive model after normalization for the *OGI* dosage effect. Genome-wide association analysis of the ability of male conversion (C) and male conversion rate (D) after normalization for the *OGI* dosage effect. The sex-chromosome (Chr. 15) was not association with either ability of male conversion or male conversion rate. The vertical red and green bars indicate the location of the association peaks. The red bars show the peaks commonly detected in the analyses using the additive and diploidized additive models, for male conversion rate. (E-G) Close-up of the association peaks on Chr. 5 (E), 8 (F), and 9 (G), with LD, using diploidized genotypes. The main haploblocks on Chr. 5, 8, and 9 included 184, 433, and 262 genes, respectively. Genotypes (Homo: homozygous, Het: heterozygous) of the most significant loci on Chr. 5 (H), Chr. 8 (I), and Chr. 9 (J).

After compensating for the effect of *OGI* allele dosage (see Materials and methods, Section 2.5 for the details), the peaks on the sex chromosome, both with quantitative and diploidized genotypes, was significantly reduced (Fig. 5A–D). Although the trends were almost identical to those in the original analysis (Fig. 4A–D), some peaks, especially those associated with the male conversion rate (Fig. 5B and D), became sharper than in the original model. Some of the original putative peaks were also reduced after compensation for the *OGI* dosage effect, presumably because their genotypes were accidentally similar to those of *OGI*. Some of the major peaks, especially on Chr. 5, 8, and 9, were common to the two models (Fig. 5A–D). On the other hand, peaks on Chr. 1, 2, and 4 were specific to additive model (Fig. 5A and B). Here, we focussed on the common candidate loci on Chr. 5, 8, and 9. To understand the combination effect of *OGI* and these three loci, multiplex regression tests were performed between the male conversion rate and the quantitative/diploidized genotypes (Supplementary Table S10), resulting in *r*^2^ values of 0.611 and 0.548 for the quantitative and diploidized genotypes, respectively. Importantly, the accuracy of the regression was significantly increased in comparison to the test without compensation for the *OGI* allele dosage ( *r*^2^ = 0.561 and 0.492 for quantitative and diploidized genotypes, respectively, Supplementary Table S9B and C). These results highlight the importance of quantitative genotyping and compensation for allele dosage in GWASs in polyploids.

Both the additive and diploidized additive models showed consistent associations between Chr. 5 and male conversion rate (Supplementary Fig. S3 and Fig. 5E, respectively). The haploblock including the peak summit was maintained over a 3 Mb region (ca 8.6–11.8 Mb), and contained 184 genes (Fig. 5E). For Chr. 8, the peak spanned the region between 3.8 and 8.8 Mb, included 433 genes (Fig. 5F), and was associated with male conversion rate in both the additive and diploidized additive models (Supplementary Fig. S4 and Fig. 5F, respectively). The quantitative genotypes for the peak summit of the Chr. 9 peak observed in the additive model (Chr9:1,287,557, Supplementary Fig. S5A) ranged from nulliplex to duplex (Supplementary Fig. S5C). The peak covered 4 Mb of the sub-telomeric region and contained 262 genes (Fig. 5G). For all three of these peaks identified from the diploidized additive model analysis, nulliplex individuals showed significantly higher male conversion rate than heterozygous individuals (Fig. 5H–J), suggesting that loss of that particular allele is associated with positive regulation of male conversion. This is consistent with the results from the additive model on Chr. 8, which indicated that fewer copies of the alternative alleles were positively correlated with male conversion rate as well (Supplementary Fig. S5B). The genotypes of the male parent ‘Taishu’ and the YTF_1_ individuals with high male conversion were consistent (recessive homozygosity) in the highest peak on Chr. 5 (Supplementary Table S11). On the other hand, the peaks of Chr. 8 and Chr. 9 showed inconsistent genotypes between the male parent ‘Taishu’ and the YTF_1_ individuals with high male conversion rate (Supplementary Table S11). These suggested that Chr. 5 might reflect the characteristics of ‘Taishu’ for male production (or *OGI* activation), whereas the other two loci might act for *OGI* activation independently.

Significant LD was maintained over ∼2–5 Mb of the peaks on Chr. 5, 8, and 9 (*r*^2^ > 0.15; Fig. 5E–G). These regions included 184, 433, and 262 genes, respectively (Fig. 5E–G and Supplementary Table S12). Next, we aimed to detect potential candidate with the genes underlying these peaks that hypothetically regulate the activation of *OGI*, which is normally silenced via SINE insertion on the promoter and the resultant DNA methylations.^11^ Considering that, in all cases, homozygous genotypes act recessively for *OGI* activation loss-of-function of genes regulating DNA/histone methylation to silence transcription would be good candidates. Histone remodelling genes such as Chromatin remodelling 4,^34^ SET domain protein 25,^35^ and histone deacetylase 19, thought to be involved in jasmonic acid and ethylene signalling in *Arabidopsis*,^36^ are all located under the Chr. 5 peak (Supplementary Table S12, Sheet1). Candidate regulators within the Chr. 8 peak included SWADEE homeodomain homolog 1,^37^ histone deacetylase 2,^38^ and Jumonji family^39^ (Supplementary Table S12, Sheet 2), while the peak of Chr. 9 included the PHD finger protein^40^ and SET domain protein 38^41^ (Supplementary Table S12, Sheet3). Future in-depth sequence analysis of these candidates might help in the identification of candidate polymorphisms potentially involved in *OGI* activation/repression.

### 3.5. Examination of GWAS in polyploid genome

Genome-wide analyses in polyploid genomes are often challenging in terms of (i) genotype calling, and (ii) haplotype phasing.^42^ For genotyping, we adopted the allele ratio per locus with high coverage for quantitative genotypes, as previously reported in potato and in blueberry.^21^^,^^43^ This approach, combined with the Pearson correlation was successful at detecting the effect of Y-chromosome dosage for *OGI* activation and other potential candidates. Furthermore, the compensation with *OGI* allele dosage with two approaches, (i) partial correlation (in correlation analysis using quantitative genotypes), and (ii) rrBLUP with the covariate of *OGI* dosage (in GWAS using diploidized genotypes), improved our ability to detect candidate loci. On the other hand, issues involving haplotype phasing still remain to be solved, since polyploids have more than two haplotypes per reference region, which are generally difficult to define, not only for conventional binary genotypes but also for quantitative genotypes. For instance, in our hexaploidy samples, the presence of many (>2) homologous haploblocks caused LD inconsistent with the order of the SNPs markers, despite of the F_1_ segregation line in which LD decay completely depends on recombinations (Fig. 5E), as observed in autotetraploid blueberry as well.^43^ In autohexaploid sweet potato (*Ipomoea batatas*) with 90 chromosomes (2*n* = 6*x* = 90), 96 linkage groups (LGs) were generated, using only double-simplex SNPs showing a Mendelian segregation ratio in an F_2_ progeny.^44^ Such construction of well-separated LGs would allow QTL analysis with conventional tools, but the targets would be limited to simplex loci and, unless the genomes were significantly different, much of the genomic space would not be included. Here, we were able to include multiplex alleles in our genome-wide association analysis, and our results suggest that a simple GWAS approach can be used to identify polysomic candidates, if sufficient markers are available.

**Figure 6:**
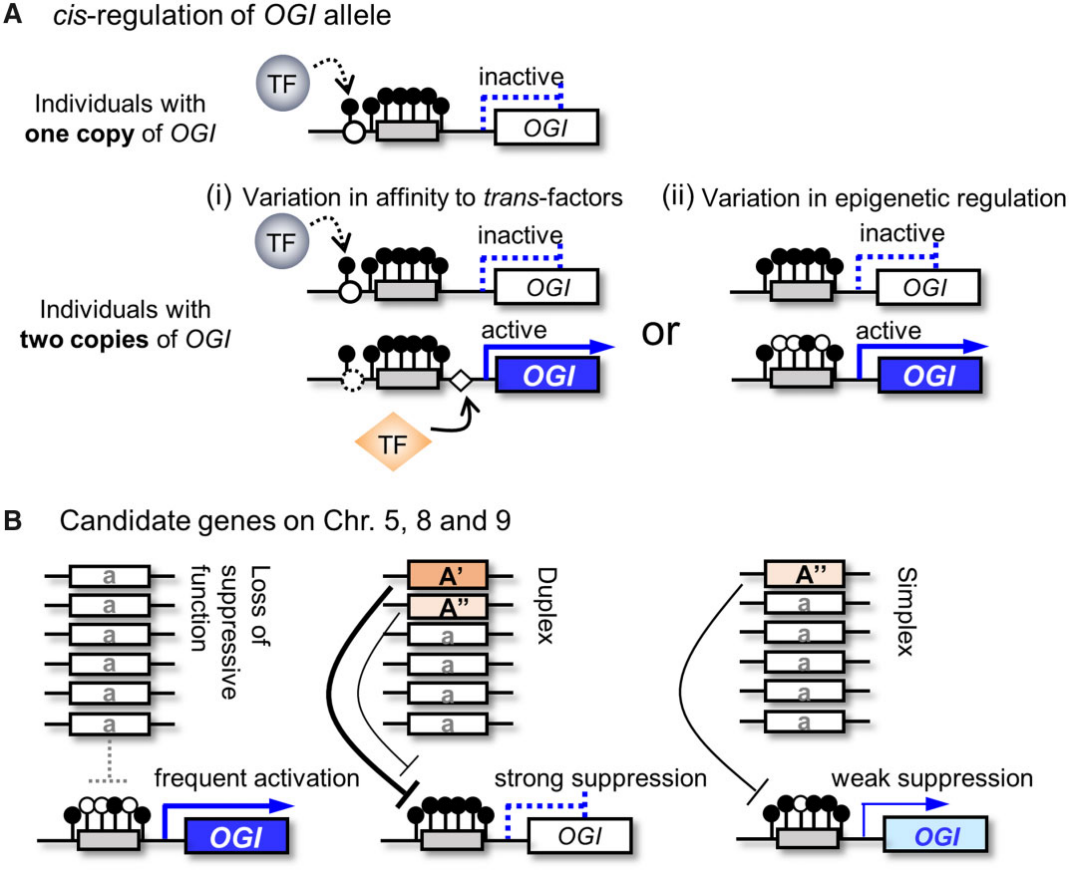
Hypothetical model for the mechanisms underlying *OGI* regulation in hexaploid persimmon. (A) Two hypothesis can be proposed for the dosage-dependent *cis*-regulation of *OGI* (see Fig. 4I): (i) variation in *cis*-factors within the *OGI* promoter sequences modulate the effect of the transcription factors accessing to the *OGI* promoter, or (ii) variation in the *OGI* promoter sequence results in a variation in epigenetic status. Both of these scenarios can be consistent with varying probability *OGI* activation, depending on the environmental conditions**. (B)** Model for the action of candidate genes on Chr. 5, 8, and 9, acting in dosage-dependent manner. Since recessive homozygosity had the highest effect on male conversion (*OGI* activation) in all three cases (Fig. 5H–J), the model predicts that the dominant (functional) alleles act to suppress *OGI*. The dosage-dependent effect (Fig. 5B) could originate either from multiple dominant alleles (A’ and A”) with slightly different ability, or additive action of a single A allele to suppress *OGI* expression.

Our results indicated that, in the additive model based on quantitative genotypes, individuals with higher dosages of *OGI* have a higher probability of male conversion. If we hypothesize that male conversion is associated with *OGI* activation, we can propose the following two hypothesis for *cis*-regulation of *OGI* to maintain *OGI* silencing (Fig. 6A): (i) multiple *trans*-acting factors can access the *OGI* promoter, each with slightly different sequence specificity, or (ii) epigenetic *cis*-factors of *OGI*, such as DNA/histone methylation, are modified independently, and thus, more copies of *OGI* alleles result in higher probability of *OGI* expression release. On the other hand, in both the additive and the diploidized additive models, all the loci on Chr. 5, 8, and 9 significantly affects male conversion rate (Fig. 5). This suggests that, although recessive alleles at these loci lack the function to maintain *OGI* suppression, the other alleles also vary in their ability to suppress *OGI* and/or have additive effects for silencing of *OGI* (Fig. 6B). Such a complex situation is not uncommon when dealing with polysomic genetic factors, and exemplifies how they can contribute to the acquisition or fine tuning of traits in a way that is not possible in a diploid situation.

## Supplementary Material

dsaa012_Supplementary_Data

## Accession numbers

The sequence reads are available from the DDBJ Sequence Read Archive (DRA) under the accession number DRA009556.
