# Supplementary material for: Genome-wide study on the polysomic genetic factors conferring plasticity of flower sexuality in hexaploid persimmon
Source: DNA Res. 2020 Jun 17;27(3):dsaa012. doi: 10.1093/dnares/dsaa012 (PMC7406971; doi:10.1093/dnares/dsaa012)
Supplement: dsaa012_Supplementary_Data [file dsaa012_supplementary_data.zip › GWASjournal_forRevision_SupplementaryTable_20200330_2.docx]

**Supplementary Table S1 Primer list for *OGI* allele detection**

**Supplementary Table S2 Data quality of ddRAD-seq in each individual**

**Supplementary Table S3 Bias in flower sexuality in 83 individuals each year**

See Excel data (Supplementary Table S3.xlsx)

**Supplementary Table S4 Percentage of male flowers produced from male parental branches**

**Supplementary Table S5 SNP loci showing significant transmission distortion (p < 1E-10)**

**Supplementary Table S6 List of loci associated with male conversion, using the additive model (A-B), and the diploidized additive model (C)**

**B**

**Male conversion rate**

**Ability of male conversion**

**A**

**Male conversion rate**

**C**

**Supplementary Table S7 *OGI* allele dosage and quantitative genotypes at chr.15:1,705,275 (sex-chr.), with male conversion rate**

Coverage for individual 20 (YTF1048) was too low at position chr.15:1,705,275 to detect the allelic ratio.

Due to the lack of DNA for five individuals (79-83), *OGI* allele dosage was estimated using quantitative genotype on chr15:17,052,751 instead (shown with *).

**Supplementary Table S8 Effect of *OGI* allele dosage on the ability to be monoecious (or ability to produce male flowers)**

*p* = 0.004

**Supplementary Table S9 Multiple regression analysis with *OGI* and other associated loci**

**A Ability of male conversion based on quantitative genotypes**

**C Male conversion rate based on diploidized genotypes**

**B Male conversion rate based on quantitative genotypes**

**Supplementary Table S10 Multiple regression analysis with *OGI*, and other loci of after normalization for the *OGI* dosage effect.**

The multiple R^2^ values significantly increased in comparison to the results without normalization for the *OGI* dosage effect (see Supplementary Table S9)

**B Male conversion rate based on diploidized genotypes**

**A Male conversion rate based on quantitative genotypes**

**Supplementary Table S11 Genotype of the parent cultivars, at the peak summit loci on Chr. 5, 8, and 9.**

**Supplementary Table S12 List of the candidate genes on chr. 5 (Sheet1), chr. 8 (Sheet2) and chr.9 (Sheet3).**

See Excel data (Supplementary Table S12.xlsx)
