# Supplementary material for: Genome-wide study on the polysomic genetic factors conferring plasticity of flower sexuality in hexaploid persimmon
Source: DNA Res. 2020 Jun 17;27(3):dsaa012. doi: 10.1093/dnares/dsaa012 (PMC7406971; doi:10.1093/dnares/dsaa012)
Supplement: dsaa012_Supplementary_Data [file dsaa012_supplementary_data.zip › GWASjournal_forRevision_SupplementaryFigure_20200330_4.doc]

**Supplementary Figure S1 Transition of male conversion rate during 5 years**

Male conversion rate tended to increase (p < 0.05 between the 1^st^ and the 2^nd^/3^rd^ years, and p < 0.01 between the 1^st^-4^th^ and the 5^th^ years)

**Supplementary Figure S2 Distribution of alternative allele ratios per individual (A) and population-wide (B)**

A. **For each individual and each SNP position, the percentage of alternative allele was recorded.**
B. **For each SNP position, the percentage of alternative allele was averaged across the entire population. Assuming random distribution of alleles, these population-wide frequencies are expected to reflect the average of the alleles present in the two parental individuals at each SNP**

**Supplementary Figure S3 Details of the association peaks identified on chr.5 using the additive model with quantitative genotypes**

A, datapoints surrounding the peak summit. B, relationship between male conversion rate and allelic ratio at the peak (chr5:8,510,403) in each individual. The quantitative genotypes (simplex or duplex) of *OGI* (chr. 15) are indicated in black and white, respectively.

**Supplementary Figure 4 Details of the association peaks identified on chr.8 using the additive model with quantitative genotypes**

A, the peaks surrounding the summit. B, relationship between the male conversion rate and the allelic ratio at the peak summit (chr8:7,052,799) in each individual. The quantitative genotypes (simplex or duplex) of *OGI* (chr. 15) are indicated in black and white, respectively.

**Supplementary Figure 5 Details of the association peaks identified on chr.9 using the additive model with quantitative genotypes**

The associated loci with ability of male conversion (A) and male conversion rate (B) (-log(p) > 4). C, relationship between the male conversion rate and the allele ratio at the summit (chr9:1,287,557) in each individual. The quantitative genotypes (simplex or duplex) of *OGI* (chr. 15) are indicated in black and white, respectively.
